# MRGD, a MAS-related G-protein Coupled Receptor, Promotes Tumorigenisis and Is Highly Expressed in Lung Cancer

**DOI:** 10.1371/journal.pone.0038618

**Published:** 2012-06-08

**Authors:** Satoko Nishimura, Makiko Uno, Yasuyuki Kaneta, Keisuke Fukuchi, Haruyuki Nishigohri, Jun Hasegawa, Hironobu Komori, Shigeki Takeda, Katsuhiko Enomoto, Futoshi Nara, Toshinori Agatsuma

**Affiliations:** 1 Biologics Research Laboratories, Daiichi Sankyo Co., Ltd., Shinagawa-ku, Tokyo, Japan; 2 Exploratory Research Laboratories II, Daiichi Sankyo Co., Ltd., Shinagawa-ku, Tokyo, Japan; 3 Biological Research Laboratories, Daiichi Sankyo Co., Ltd., Shinagawa-ku, Tokyo, Japan; 4 Oncology Research Laboratories, Daiichi Sankyo Co., Ltd., Edogawa-ku, Tokyo, Japan; 5 Department of Chemistry and Chemical Biology, Gunma University Graduate School of Engineering, Gunma, Japan; 6 Department of Molecular Pathology and Tumor Pathology, Akita University Graduate School of Medicine, Akita, Japan; 7 Cardiovascular-Metabolics Research Laboratories, Daiichi Sankyo Co., Ltd., Shinagawa-ku, Tokyo, Japan; Sun Yat-sen University Medical School, China

## Abstract

To elucidate the function of MAS-related GPCR, member D (MRGD) in cancers, we investigated the *in vitro* and *in vivo* oncogenic function of MRGD using murine fibroblast cell line NIH3T3 in which MRGD is stably expressed. The expression pattern of MRGD in clinical samples was also analyzed. We found that overexpression of MRGD in NIH3T3 induced focus formation and multi-cellular spheroid formation, and promoted tumors in nude mice. In other words, overexpression of MRGD in NIH3T3 induced the loss of contact inhibition, anchorage-independent growth and *in vivo* tumorigenesis. Furthermore, it was found that the ligand of MRGD, beta-alanine, enhanced spheroid formation in MRGD-expressing NIH3T3 cells. From investigation of clinical cancer tissues, we found high expression of MRGD in several lung cancers by immunohistochemistry as well as real time PCR. Based on these results, MRGD could be involved in tumorigenesis and could also be a novel anticancer drug target.

## Introduction

G protein-coupled receptor (GPCR) family members activate various physiological signaling and play an important role in the development as well as function of each organ [1]. In addition, diverse GPCRs have been found to be overexpressed in primary and metastatic tumor cells of head and neck squamous cell carcinoma, non-small cell lung cancer, breast, prostate and gastric tumors, melanoma and diffused large B cell lymphoma [2]. Some GPCRs have also been reported to be functionally involved in the cancer progression [3], such as gastrin-releasing peptide receptor (GRPR) in prostate cancer [4], CXCR4 in metastasis [5] and so forth. MAS1, is the first GPCR to be reported to have any relation to cancer development. It was reported that NIH3T3 cells ectopically expressing MAS1 promoted focus formation *in vitro* and facilitated tumorigenesis in nude mice [6], however, neither significant MAS1 expression nor active MAS1 mutation have been reported in clinical cancers, therefore, the role of MAS1 in cancer is still unclear. On the other hand, high expression of MAS1 was observed in the central nervous system, such as hippocampus and cerebellum, and MAS1 enhanced the ligand-dependent calcium influx of Ang II receptor (AT2R) where MAS1 formed a complex with AT2R. These suggest that MAS1 plays an important role in the central nervous system [7], [8].

MAS-related G-protein coupled receptor, D (MRGD), also referred to as hGPCR45 [9] or TGR7 [10], was identified as a novel GPCR in murine and human genomes [11]. It was found that MRGD serves as the receptor of beta-alanine [12]. Several MRG family members were reported to be expressed in specific subpopulations of sensory neurons, which detect pain stimuli [11]. As for MRGD, its expression was found in dorsal root ganglia (DRG) and co-localized with Vanilloid receptor-1 (VR-1), which is an essential receptor for heat and pain sensation [12]. Moreover, genetic ablation of MRGD expressing neuron reduces behavioral sensitivity to noxious mechanical stimuli but not to heat or cold stimuli in mice [13]. Thus, MRGD is considered to be one of the players in pain sensation and/or transduction. It was also reported that MRGD transduces intracellular signaling of the angiotensin (Ang) II metabolite, Ang-(1–7) [14]. As described above, the function of MRGD in the central nervous system has been observed by several groups.

There are several GPCR family members showing amino acid sequence similarity to MAS1 such as MRGA, MRGB, MRGC, MRGD, MRGE, MRGF, MRGG, MRGH and MRGX [11]. In the phylogenic tree of the MRG family, MAS1, MRGD, MRGE, MRGF and MRGH are categorized as belonging to the same branch [11]. This raised the hypothesis that the genes in the phylogenic branch including MAS1 could have a similarity in function or signal transduction. We noticed the ability of MAS1 to promote tumorigenic function in NIH3T3, and in this study, attempted to elucidate the tumorigenic function of MRGD, which is reported to work in the central nervous system such as MAS1. We also investigated the expression of MRGD in human cancer tissues. We found that MRGD promotes the loss of contact inhibition, anchorage-independent growth and *in vivo* tumorigenesis and is also highly expressed in several human lung cancers, suggesting that MRGD could play an important role in human cancer.

## Results

### Effect of MRGD on cell proliferation and tumorigenicity in vitro

**Figure 1:**
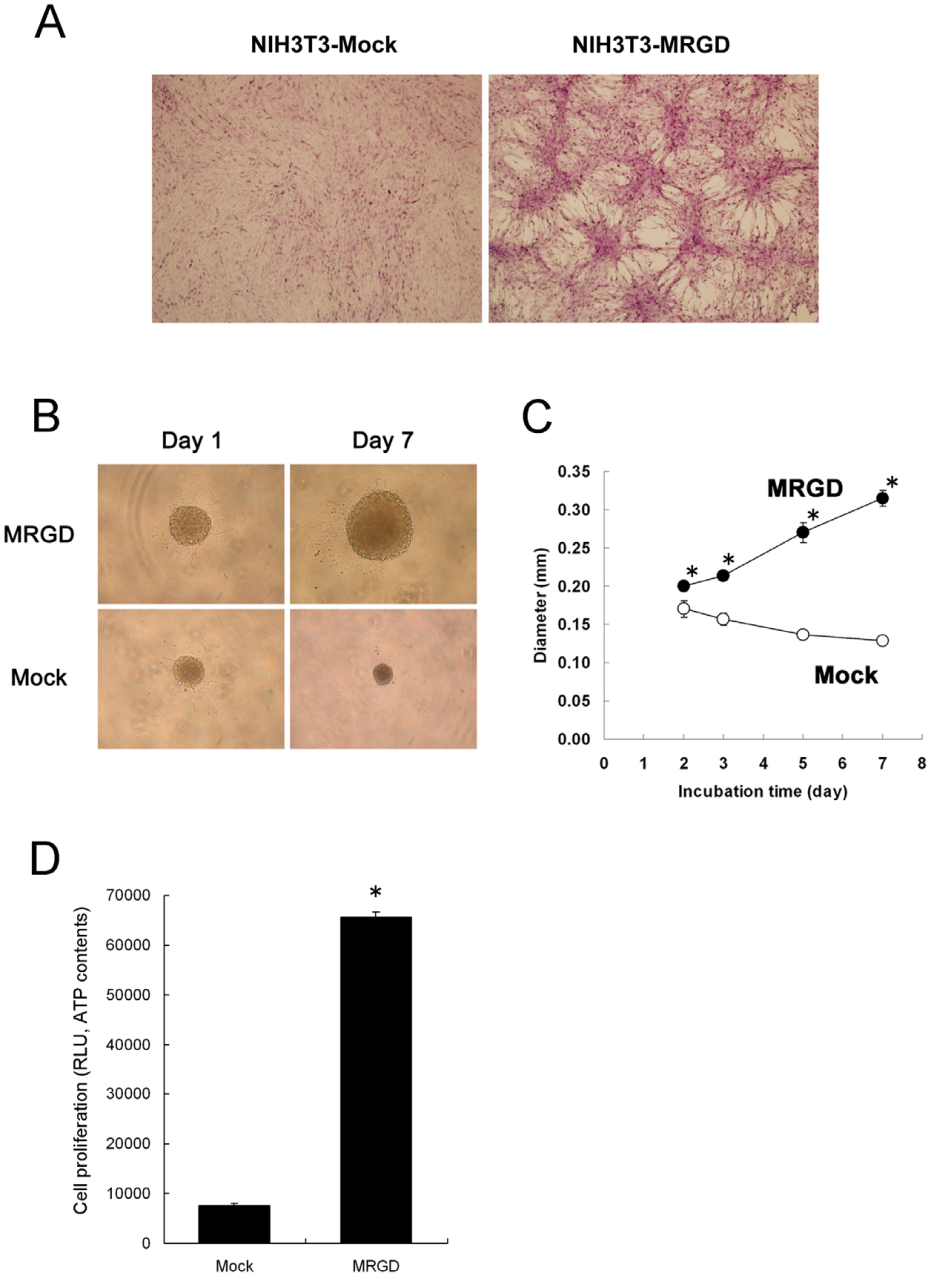
Tumorigenesis of NIH3T3 cells stably expressing MRGD (NIH3T3-MRGD cells). **A.** Representative pictures of focus formation in monolayer cultures of NIH3T3 cells stably expressing Mock (NIH3T3-Mock cells, left) or MRGD (NIH3T3-MRGD cells, right). Cells were stained with crystal violet after fixing with 4% paraformaldehyde. **B.** Representative pictures of NIH3T3-MRGD or NIH3T3-Mock spheroid on Days 1 and 7. **C.** Spheroid growth curves. The spheroid sizes of NIH3T3-MRGD (closed) or NIH3T3-Mock (open) at Days 2, 3, 5 and 7, are shown with their diameters (mean ± SD). * indicates p<0.005 (Mann-Whitney U test, 2 tails). **D.** Cell proliferation of spheroid cultures of NIH3T3-MRGD or NIH3T3-Mock. Luminescence of whole cell ATP contents (means ± SD) was measured at 6 days after plating. * indicates p<0.005 (Mann-Whitney U test, 2 tails).

To clarify the effect of MRGD on cell growth, the NIH3T3-MRGD cell line, which NIH3T3 cells were stably transfected with MRGD retroviral expression vector was established and its growth-related profiles were analyzed. The gene expression of MRGD in the NIH3T3-MRGD cell line was confirmed by RT-PCR and sequencing (Figure S1). Using the NIH3T3-MRGD cells, the focus formation assay (see Materials and Methods) was performed, where significant foci formation was seen in the NIH3T3-MRGD cell culture, while no such foci were seen in NIH3T3-Mock (Figure 1A). These data indicate that MRGD gene expression cancels contact inhibition of NIH3T3 cells, one of the features of normal fibroblasts. To determine MRGD's other growth-related features, the spheroid growth assay (see Materials and Methods) was performed, where NIH3T3-MRGD cells and NIH3T3-Mock cells were cultured on a 96-well non-adherent U-bottomed plate, respectively. First, we noticed that larger spheroids were observed for NIH3T3-MRGD than for NIH3T3-Mock on the 5th day after plating. The spheroid of NIH3T3-Mock shrunk during cultivation, while that of NIH3T3-MRGD obviously grew day by day, as shown in Figure 1B. We determined this growth-related character by measuring change in the diameters of the spheroids and also by measuring difference in ATP activity of the spheroids (Figure 1C and D). Significantly larger spheroid diameters were observed for NIH3T3-MRGD than those for NIH3T3-Mock from 2 to 8 days after plating (Figure 1C, p<0.005, Mann-Whitney U test, 2 tails). Also, much more ATP content was seen for NIH3T3-MRGD compared to that of NIH3T3-Mock at 6 days after plating (Figure 1D, p<0.005, Mann-Whitney U test, 2 tails). Similar results were obtained by [^3^H]-Thymidine incorporation assay measurement (Figure S2, File S1). These results indicate that MRGD significantly promotes anchorage-independent growth of the normal fibroblast, in other words, MRGD possess oncogenic capability.

### *Effect of MRGD on cell proliferation and tumorigenicity in vivo*

Next, to evaluate *in vivo* tumorigenic activity, we subcutaneously inoculated NIH3T3-MRGD or NIH3T3-mock to athymic nude mice. The remarkable growth of grafted tissue was observed with the mice subcutaneously implanted with NIH3T3-MRGD cells but not with NIH3T3-Mock cells (Table 1). The average tumor volumes of NIH3T3-MRGD-cells-grafted mice exceeded 2,000 mm^3^ in 21 days after inoculation. In contrast, no remarkable growth was seen in NIH3T3-Mock-cells-grafted mice until 21 days after inoculation. A clump of cells was found in NIH3T3-Mock-cells-grafted mice 24 days after inoculation, however, it was still very small and its average volume was less than 400 mm^3^. As NIH3T3-MRGD formed the large clump *in vivo*, we performed HE staining to confirm that the NIH3T3-MRGD clump possessed tumor-like pathological features. HE staining of the grafted tissue sections of NIH3T3-MRGD-cells-grafted mice showed fibrosarcoma-like morphology (Figure S3). Taken together, this result suggests that MRGD expression causes not only *in vitro* but also *in vivo* tumorigenicity.

**Table 1:** Tumor volumes of NIH3T3-MRGD or Mock bearing mice.

Days	Tumor Volume (mm^3^)					
	Mock			MRGD		
3	0.0	0.0	0.0	0.0	0.0	0.0
5	20.1	16.1	58.4	57.8	41.5	130.9
7	70.3	18.5	31.6	161.1	159.2	184.0
10	0.0	10.4	0.0	469.9	314.3	596.4
12	39.3	17.5	0.0	584.0	669.0	859.1
14	58.8	11.8	4.7	1070.6	2166.4	1162.4
17	54.7	11.5	0.0	2548.3	2831.5	1840.6
19	124.2	25.3	110.7	2642.3	3709.0	1974.3
21	124.1	26.0	83.0	2400.4	4409.7	2403.5
24	94.3	380.8	284.2	3722.6	4562.6	2730.5
26	854.2	94.3	495.3	3998.9	5155.4	3292.1

Tumor volumes (mm^3^) were calculated according to the following equations: Tumor volume (mm^3^) = 1/2×(tumor length)×(tumor width)^2^

### Induction of cell growth by ligand stimulation

**Figure 2:**
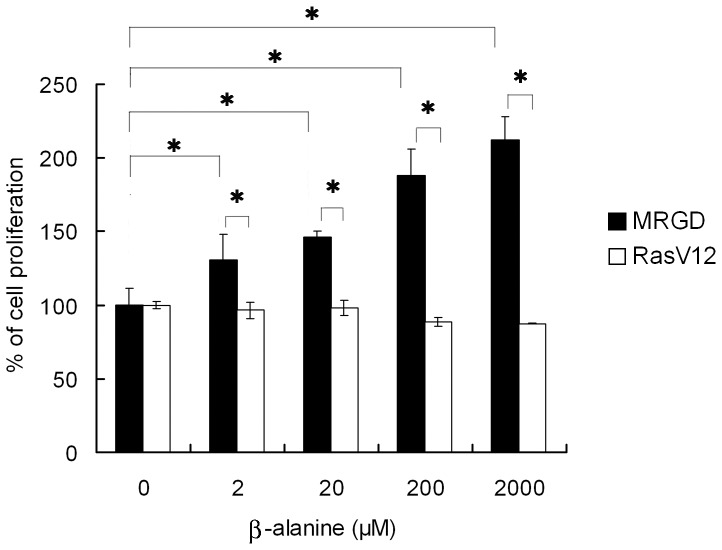
MRGD ligand, beta-alanine, promoted cell proliferation of NIH3T3-MRGD cells. NIH3T3 cells transfected with MRGD or RASV12 were cultured in RPMI1640 containing 0.1% BSA and various concentration of beta-alanine for 7 days. Data were obtained from three independent cell cultures (means ± SD). * indicates p<0.05 (Mann-Whitney U test, 2 tails), compared with NIH3T3-MRGD without beta-alanine, and with NIH3T3-RASV12.

To evaluate the effect of beta-alanine, one of the MRGD ligands [12], on spheroid growth promoted by MRGD, beta-alanine in various concentrations was added to the spheroid culture of NIH3T3-MRGD cells or NIH3T3-RASV12 cells. NIH3T3-Mock cells did not grow well in spheroid culture and no stable growth was induced even in the presence of beta-alanine (data not shown), and therefore, the NIH3T3-Mock cell group was not set in this study. In this experiment, significant promotion of cell growth by beta-alanine in a dose-dependent manner was observed for NIH3T3-MRGD as measured by ATP activity (p<0.05, Mann-Whitney U test, 2-tails), while no effect of beta-alanine was seen for NIH3T3-RASV12 spheroid growth even with 2000 µg/ml of beta-alanine (Figure 2). These data indicate that anchorage-independent growth of MRGD expressing cells can be enhanced by its ligand stimulation.

### Expression of MRGD in human cancers

**Figure 3:**
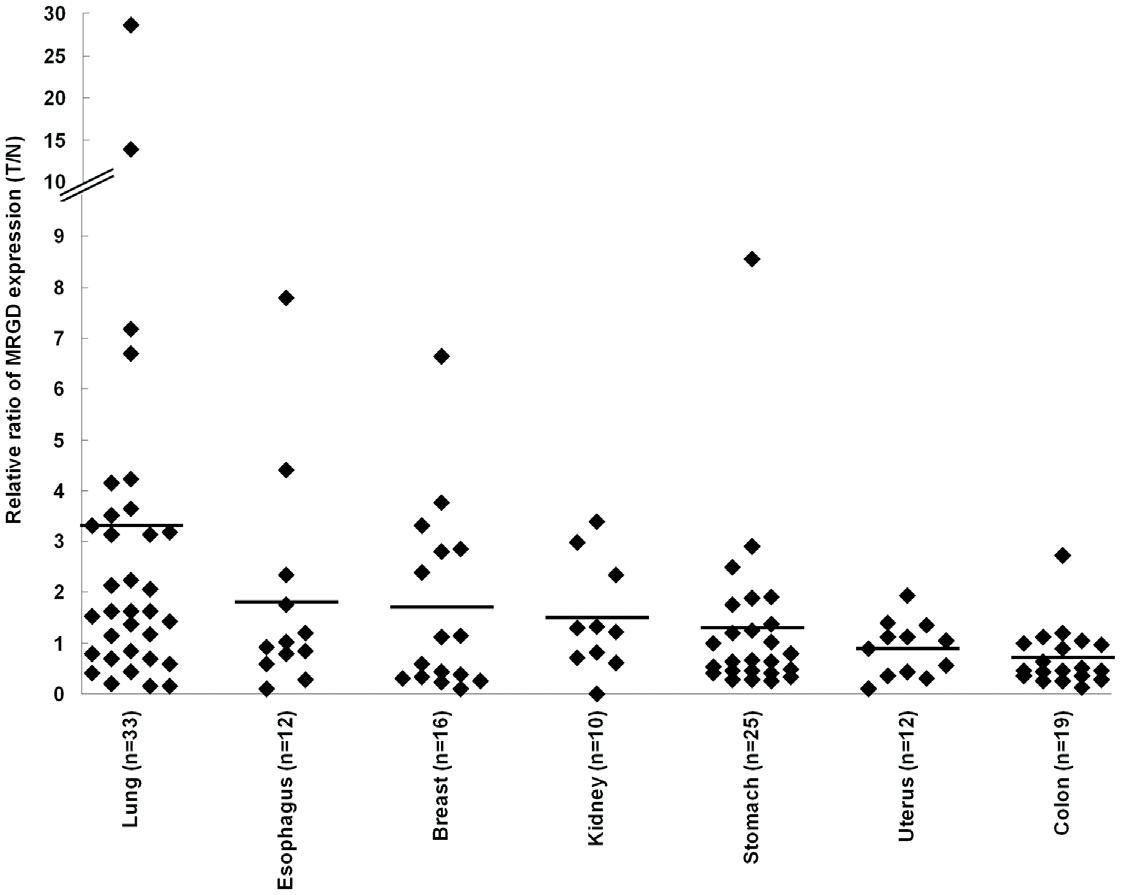
IHC analysis of MRGD expression. Cell block samples were used to confirm the antibody specificity in immunostaining. HEK293/αvβ3 cells transfected with MRGD expression vector (**A**) or Mock vector (**B**) are shown. **C.** The representative staining of lung adenocarcinoma which is positive for MRGD.

We determined the MRGD expression in several clinical cancers. First, to clarify the MRGD protein expression level in lung cancer samples, we performed IHC using a pair of tumor and normal tissue sections of 33 pairs of human lung cancer samples (see Materials and Methods). For IHC, the rabbit anti-MRGD antibody was raised and antigen specificity of the antibody was confirmed as shown in Materials and Methods; staining signals of the anti-MRGD antibody at the outer membrane were detected in formalin-fixed HEK293/αvβ3 cells transfected with MRGD, although not in Mock-transfected HEK293/αvβ3 cells (Figure 3A and B). With this antibody, 22 out of 33 clinical lung cancer cases showed MRGD positive signals (22/33): adenocarcinomas (9/10), poorly-differentiated squamous cell carcinomas (7/10) and well-differentiated squamous cell carcinomas (6/10) (Table 2). We noticed especially strong signals in some samples, including adenocarcinomas (8/9) (Figure 3C and S4), poorly-differentiated squamous cell carcinomas (3/7) and well-differentiated squamous cell carcinomas (2/10). On the other hand, no staining signal was detected for small cell carcinoma (date not shown).

**Table 2:** IHC analysis of MRGD expressions in lung cancer.

Histological type	Number of cases		
	Total	MRGD positive	(%)*
Adenocarcinoma	10	9	(90)
Poorly-differentiated squamous cell carcinoma	10	7	(70)
Well-differentiated squamous cell carcinoma	10	6	(60)
Small cell carcinoma	3	0	(0)

* % was calculated on MRGD positive cases/total cases.

**Figure 4:**
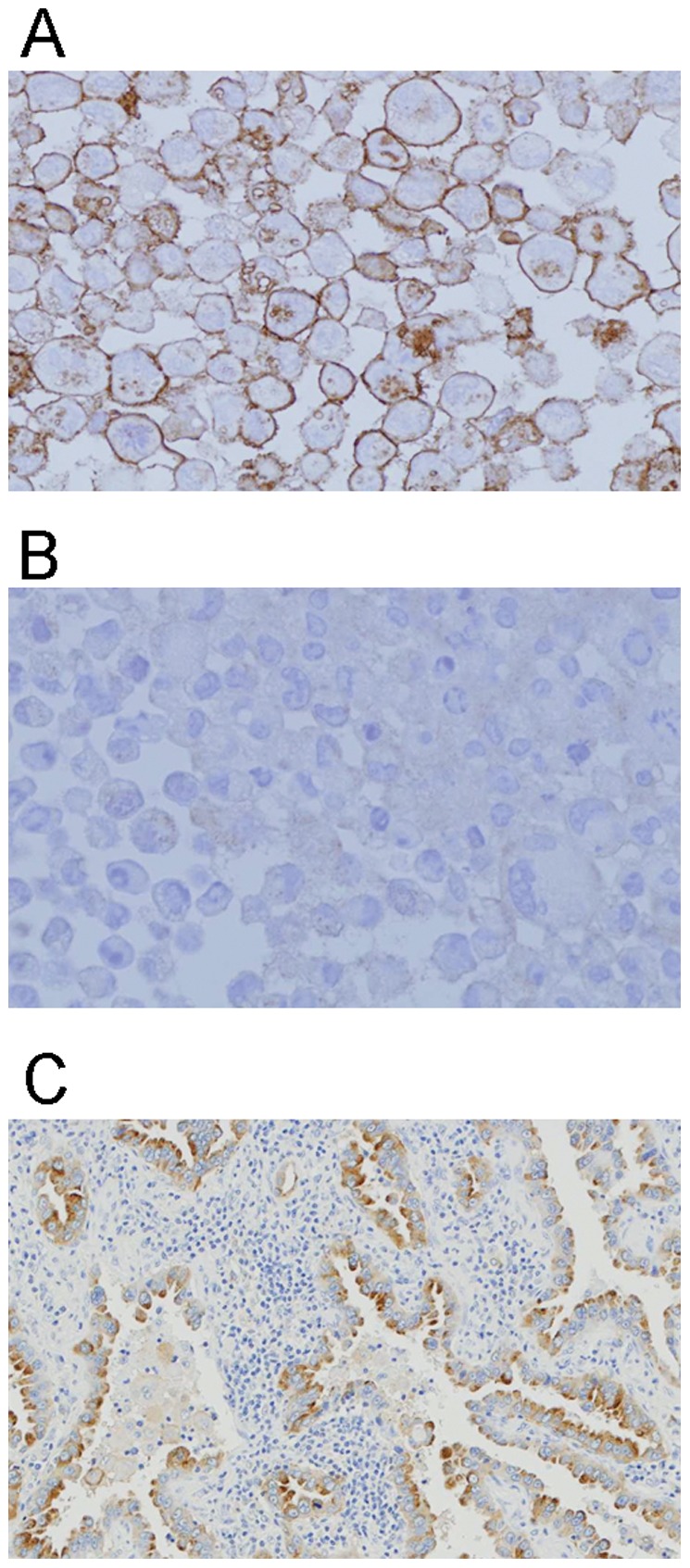
Quantitative RT-PCR analysis of MRGD mRNA expression in clinical samples. One hundred and twenty seven sets of RNA samples of cancer and non-cancer portions from the same patients with lung (n = 33), esophagus (n = 12), breast (n = 16), kidney (n = 10), stomach (n = 25), uterus (n = 12) or colon (n = 19) cancer. Ratios of the amount of MRGD mRNA in tumor portion per that in normal portion of each case were plotted. The bar indicates the mean of the ratio in each cancer type.

We also analyzed MRGD gene expression in clinical cancers by quantitative RT-PCR. One hundred and twenty seven sets of RNA samples with both cancer and non-cancer portions of lung, esophagus, breast, kidney, stomach, uterus and colon cancer tissues were used. In the samples of uterus or colon tissue, MRGD expression in the cancer portion never exceeded 3 times the amount in the normal portion. As for lung cancers, the average of MRGD expression in the cancer portion exceeded the amount equal to 3 times as much as that in the lung normal portion, and for 12 out of 33 lung pair samples, the MRGD expression in the cancer portion exceeded 3 times the amount in the paired normal portion (Figure 4). In all 7 cancer species determined here, the lung pair samples showed the highest frequency (36%) for higher MRGD expression in the cancer portion compared to that of the normal portion with the criteria exceeding 3 times the amount (Figure 4). Some other cancer samples, such as breasts (3 out of 16), esophagi (2 out of 12), kidneys (1 out of 10) and stomachs (1 out of 25), also showed 3 times higher expression in the cancer portion compared to that in the normal portion. We did not see a statistical difference in the overall comparison of the mRNA signal between normal and cancer portions in each cancer type by one-way ANOVA. More precise categorization of the patients must be necessary to obtain statistical significance. However, the data clearly indicate that over 3-fold higher expression signals are shown in the tumor rather than the normal portion in some cancer patients. Higher expression of the cancer portion in lung cancer was also very consistent with the results of IHC. These suggest that the data are very meaningful to indicate the next direction of research on MRGD expression in cancer.

## Discussion

We demonstrated that the MRGD expression induces loss of contact inhibition, anchorage-independent spheroid growth *in vitro* and also tumorigenesis *in vivo*, which are not seen in the parental normal fibroblast cells (Figure 1, Table 1). These in functional phenotypes observed for MRGD are quite similar to those reported for a representative oncogene, RASV12 [15]. We also confirmed that RASV12 expression promotes the cancellation of contact inhibition of normal fibroblast cells and also induces spheroid growth or anchorage-independent cell growth (data not shown). Moreover, we showed that NIH3T3-MRGD cells resulted significant growth of fibrosarcoma-like cells *in vivo* (Figure S3), and their morphological phenotypes were quite similar to the NIH3T3-RASV12 cells (data not shown). These results strongly support that MRGD transduces tumorigenic signaling and promotes anchorage-independent cell growth seen with RASV12. It was reported that iPS cells was made from mouse embryonic fibroblast (MEF) reprogrammed by introducing several genes including oncogenes [16]. In this study, we focused on the tumorigenic function of MRGD, however, it might be also of interest to clarify whether or not MRGD is able to promote stemness. The expression of stemness markers such as Oct-3/4, Nanog and so forth [16]–[19] in NIH3T3-MRGD and other MRGD-positive cells, as well as the expression of MRGD itself in stem cells and reprogrammed MEFs, are of interest to know the importance of MRGD in this subject and should be elucidated in the future.

A number of reports indicate that not only oncogene such as RASV12 but also proto-oncogenes such as ERBB2 and FYN give similar tumorigenic phenotype in fibroblast cells [20], [21]. Additionally, the expression of such oncogenes and/or proto-oncogenes is often increased in various human cancers and that their cell growth and anti-apoptotic signals promote not only cancer growth but also disease progression in cancer patients [2], [22]–[25]. In this report we found that MRGD mRNA is expressed in clinical human cancer samples from some patients with lung, breast, esophagus, kidney or stomach cancer. In addition, we found that some of those cancer samples expressing MRGD mRNA showed relatively higher MRGD protein expression compared to the non-cancer portions from the same patients, although no overall statistical significance was seen in each tumor type (Figure 4). For lung cancers, our RT-PCR analyses showed high and frequent MRGD mRNA expression, and our IHC analyses revealed that the highest level, which we detected for MRGD protein expression, was observed frequently in human lung cancers (Table 2), especially in lung adenocarcinomas (Figure 3). Although further analyses of MRGD functions in human cancer cells and tissues are needed, the expression profile of MRGD in clinical cancers revealed in this study strongly suggests the possible contribution of the oncogenic function of MRGD itself and/or a related signal pathway in some solid tumors such as lung cancers. GPCRs could be good targets of small molecule inhibitors as well as antibodies, and therefore MRGD, a GPCR, could serve as a novel target for cancer therapy. Lung adenocarcinomas might be an especially interesting cancer type for possible anticancer therapy targeting MRGD.

Mechanisms by which MRGD promotes oncogenic signals remain unknown. However, our results indicate that MRGD expression promotes oncogenic phenotypes in normal cells both *in vitro* and *in vivo* (Figure 1, Table 1), and also that the ligand, beta-alanine, activates MRGD-dependent cell growth *in vitro* (Figure 2). It was reported that normal levels of beta-alanine was about 3.8 µM in healthy human plasma [26]. Also reported, was that beta-alanine concentrations in nerve-related tissue are shown to be about 50 µM in rat sciatic nerve and 60 µM in cat brain [27], [28]. Our data indicate that beta-alanine promotes spheroid growth from 2 µM to 2000 µM in a dose dependent manner, and 50% growth promotion was observed at 20 µM (Figure 2). Therefore, beta-alanine in healthy human plasma is high enough to promote spheroid growth via MRGD. A report has to date, revealed that high beta-alanine concentration is linked to cancer; in detail, mammary tumors of rat or mouse contain high level beta-alanine which have never been found in normal mammary tissue of rat or mouse [29]. Hence, beta-alanine concentration in plasma or tumors of cancer patients might be higher than that of normal subjects. It might be possible that the beta-alanine activates tumor growth and survival signals in cancer patients through MRGD to some extent. Additionally, in cancer tissues, high MRGD expression may cause constitutive transduction of oncogenic signals, or may cause higher ligand responsiveness than in normal tissues which leads to promotion of cancer growth. On the other hand, no report has revealed that beta-alanine promotes cancer development, and therefore, it is possible that there might be another MRGD ligand contributing to cancer development through MRGD. These should be elucidated in the future.

In this study, we demonstrated; 1) tumorigenic activity of MRGD *in vivo* and *in vitro*, 2) MRGD expression in clinical cancer tissues, 3) promotion of spheroid growth by beta-alanine, the MRGD ligand, and 4) fibrosarcoma-like morphology of the grafted tissues of mice subcutaneously implanted MRGD-expressing cells. These suggest that MRGD is a potent target in cancer therapy and that small molecule antagonists, antibodies or RNAi for MRGD would provide promising anticancer therapy.

## Materials and Methods

Written informed consent was received from all participants when we obtained all clinical tissues, and all the experiments of the clinical tissues were carried out according to protocols approved by Daiichi Sankyo research ethics committee. All animal work has been conducted according to the relevant national guideline in 2005, when the animal works were studied.

These animal experiment protocols were approved by Sankyo animal research ethics committee, in Sankyo Co., Ltd., which was the antecedent of Daiichi Sankyo Co., Ltd.

### Materials and cell culture

FBS was purchased from Hyclone (South Logan, UT). DMEM, RPMI1640, Opti-MEM, Geneticin and Lipofectamine 2000 were purchased from Life Technologies (Carlsbad, CA). Retrovirus packaging cell line, 293-10A1, was established in our laboratory from 293 cell line (ATCC, CRL-1573) by transfecting pCL-10A1 (IMGENEX, Sorrento Valley, CA). The 293-10A1 cell line was maintained with DMEM medium supplemented with 10% FBS, 3 µg/ml of blasticidin (Wako, Osaka, Japan) under conditions of 5% CO_2_ at 37°C. NIH3T3 cell line was obtained from ATCC (CRL-1658) and was adjusted for RPMI1640 medium supplemented with 10% FBS. Hexadimethrine bromide was purchased from Sigma-Aldrich (Tokyo, Japan). HEK293/αvβ3 cell line was established from 293 cells (ATCC, CRL-1573) by stable transfection with integrin αv and β3 expression vectors, and was cultured in DMEM medium supplemented 10% FBS.

### Generation of NIH3T3-MRGD cells

cDNA encoding full-length human MRGD, RASV12 or GFP was cloned into a pLNCX vector (Clontech Laboratories, Mountain View, CA). For infection, NIH3T3 cells were seeded in a 10 cm dish and cultured for 1–3 days. The 293-10A1 cells were plated on a 10 cm collagen I-coated dish (AGC Techno Glass, Chiba, Japan) and were cultured overnight. pLNCX-MRGD, pLNCX-RASV12 or pLNCX-Mock was transfected into the 293-10A1 cells by Lipofectamine 2000. The supernatant of the transfected 293-10A1 cells were collected, then fresh medium were added for the next cycle. The collected supernatant was filtrated with a 0.45 µm PVDF membrane (Millipore, Billerica, MA) and the equal volume of RPMI1640 medium supplemented with 10% FBS and 16 µg/ml Hexadimethrine bromide were added. This viral solution was added to NIH3T3 cells for infection. The series of these infection procedures were repeated three times every 12 hours. After 12 hours from the last infection, the cells were cultured with 500 µg/ml of Geneticin for one week to accumulate cells expressing the target gene.

### Focus formation assay

Cells were plated at 1×10^5^ cells/well on a 6-well cell culture plate (Corning Japan, Tokyo, Japan) and cultured for one week. The cultured cells were fixed with 4% paraformaldehyde (Wako, Osaka, Japan) for 30 minutes at 4°C and were stained with 0.5% crystal violet (Sigma-Aldrich Japan, Tokyo, Japan).

### Spheroid growth assay

NIH3T3-MRGD cells were suspended in RPMI1640 medium supplemented with 10% FBS or 0.1%BSA and plated on 5000 cells/well in 100 µl on a 96-well spheroid plate (Sumitomo Bakelite, Tokyo, Japan). In the case where beta-alanine was added, cells were plated on 5000 cells/well in 80 µl and then 20 µl of beta-alanine was added on the same day. Spheroid growth was measured with any of the following methods. 1) Measuring spheroid diameter: Spheroid diameter was measured by ocular micrometer. Each diameter was calculated from the magnification of an objective lens and an eyepiece. 2) Measuring spheroid's ATP quantity: Cell Titer-Glo Luminescent Cell Viability Assay (Promega KK, Tokyo, Japan) was used according to the manufacturer's instructions. Then, spheroid with 100 µl of reagent was well pipetted and transferred to a white flat bottom plate (Corning Japan, Tokyo, Japan). The 1 sec luminescence of the plate was measured by Mithras LB940 (Berthold Technologies, Wildbad, Germany).

### Analysis of in vivo tumorigenicity of MRGD-transfected NIH3T3 cells

Athymic nude mice (BALB/cAJcl-nu/nu mice, CLEA Japan, 5 weeks old, female) were inoculated subcutaneously with 3×10^6^ NIH3T3-MRGD cells (n = 3) or NIH3T3-Mock cells (n = 3), as a negative control. Tumor volume was measured 3 times per week. The tumor volumes were calculated according to the following equations: 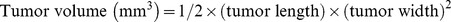
According to the relevant national guideline, all mice were to be euthanized if sign of the toxicity such as reduced activity, reduced appetite, reduced drinking, licks, guards limbs, increased aggression, vocalization, aversion toward con-specifics, dehydration, missing anatomy, abnormal posture, fractured appendage and prolapse, diarrhea, progressive dermatitis, hunched posture, lethargy or persistent recumbency, coughing, labored breathing, nasal discharge, jaundice and/or anemia, neurological signs, bleeding from any orifice, self-induced trauma, difficulty with ambulation, excessive or prolonged hyperthermia or hypothermia, or weight loss which exceeded 10% of total body weight of negative control mice, was seen. No sign of toxicity was seen in the mice during this experiment.

### Preparation of rabbit anti-human MRGD polyclonal antibody

The mixture of 2 kinds of peptides, GTVESALNYSRGSTVH (16 mer) and ELEGGETPTVGTNEMGA (17 mer), was used as an antigen. Serums were obtained from rabbits immunized with the antigen prepared with incomplete Freund's adjuvant (DIFCO, Detroit, MI) except for the first immunization, which contained complete Freund's adjuvant (DIFCO, Detroit, MI). Finally, polyclonal IgG antibodies were purified from the serums by an affinity column with the antigen peptides.

### Immunohistochemistry (IHC)

The specificity of anti-MRGD antibodies were validated by staining HEK293/αvβ3 cells transfected with MRGD-expressing or empty vector. These transfectants were fixed with 20% formalin neutral buffer solution (Wako, Osaka, Japan) and paraffin embedded to make cell blocks. The cell blocks were cut out to thin sections by microtome and set on an APS-coated slide glass. The sections were dried, de-parafinized and pretreated by Biotin Blocking System (Dako Japan, Tokyo, Japan). The sections were then blocked with protein block serum-free (Dako Japan, Tokyo, Japan) and were treated with a polyclonal anti-human MRGD antibody (1/200) in antibody diluent (Dako Japan, Tokyo, Japan). The antibody-treated sections were treated with biotinylated anti-rabbit IgG, then treated with ABC-AP of Vectastain ABC alkaline phosphatase kit rabbit IgG (CliniScience, Montrouge, France). AP red of Alkaline Phosphatase Substrate Kit I was used as a coloring substrate. Cancer tissue sections were prepared from paraffin embedded blocks and immunohistochemical staining was performed with the ABC method.

### Measurement of MRGD gene expression in clinical tissue

Total RNA of tumor or non-tumor clinical tissues from the same donor were purchased from BioChain (Hayward, CA). These RNA pair samples were treated with ATP-Dependent DNase (Toyobo, Osaka, Japan) with 10 units of RNase inhibitor (Toyobo, Osaka, Japan) for 15 minutes on ice. The DNase was removed by the total RNA miniprep system (VIOGENE, Taipei County, Taiwan). To obtain cDNA, a mixture of template total RNA (2 ng), random primers (9 mer, 50 pmol), ReverTra Ace (10 units, Toyobo, Osaka, Japan) and reaction buffer (50 mM Tris-HCl pH 7.5, 75 mM KCl, 6 mM MgCl_2_, 1 mM DTT, 1 mM dNTP buffer) was incubated at 25°C for 15 minutes, 42°C for 30 minutes, and then 95°C for 3 minutes. Real time-PCR reaction was performed with TaqMaq One-Step RT-PCR Master Mix (Life Technologies Japan, Tokyo, Japan) in accordance with manufacturer's instructions using the following probe and primers.

*MRGD*-2 (TaqMan probe sequence): TGTGTGCCACCATGCCTGGCTAATT

*MRGD*-F2 (Forward primer sequence): GCTCACTACAACCTCAATGTGCC

*MRGD*-R2 (Reverse primer sequence): GCCACATAGCAAGATCTCATCTCTAC

Data were normalized by TaqMan β-actin control reagent (Life Technologies Japan, Tokyo, Japan). These gene expressions were measured using Mx3000P QPCR System (Agilent Technology, Santa Clara, CA).

### Statistical Analyses

All the *vitro* experiments were analyzed by Mann-Whitney U-tests, except quantitative RT-PCR analysis in clinical samples, which were analyzed by one-way ANOVA. Reported p values are two-tails and are considered to be statistically significant at p<0.05.

## Supporting Information

Figure S1 **Agarose gel electrophoresis of PCR-amplified cDNA inserts.** Nucleic acid sequences of PCR products for vector inserts were verified. Lane 1 and 3: RT-PCR-amplified GFP from total RNA of NIH3T3-GFP; lane 2: Amplified GFP from GFP plasmid (positive control); lane 4 and 6: RT-PCR-amplified MRGD from total RNA of NIH-3T3-MRGD; lane 5: Amplified MRGD from MRGD plasmid (positive control).
(TIF)

Figure S2 **Cell proliferation of NIN3T3-MRGD spheroid measured by [^3^H]-Thymidine incorporation.** [^3^H]-Thymidine incorporation into the spheroids was measured at 6 days after plating. * indicates p<0.005 (Mann-Whitney U test, 2 tails).
(TIF)

Figure S3 **HE staining of NIH3T3-MRGD grafted tissue in nude mouse.** The NIH3T3-MRGD clumps on 7 days after inoculation in athymic mice (A, ×40, B, ×800) showed a cellular representative spindle cell tumor tissue type.
(TIF)

Figure S4 **HE and IHC stainings of lung adenocarcinoma samples with anti-MRGD antibody.** HE staining (**A**, **C**, **E**) and IHC staining (**B**, **D**, **F**) were performed. These are the examples from three independent patients with lung adenocarcinoma. Patient 1, **A**, **B**; Patient 2, **C**, **D**; Patient 3, **E**, **F.**
(TIF)

File S1 **Material and Methods of Figure S2.**
(DOC)
