# Supplementary figures and images for: MRGD, a MAS-related G-protein Coupled Receptor, Promotes Tumorigenisis and Is Highly Expressed in Lung Cancer
Source: PLoS One. 2012 Jun 8;7(6):e38618. doi: 10.1371/journal.pone.0038618 (PMC3370999; doi:10.1371/journal.pone.0038618)

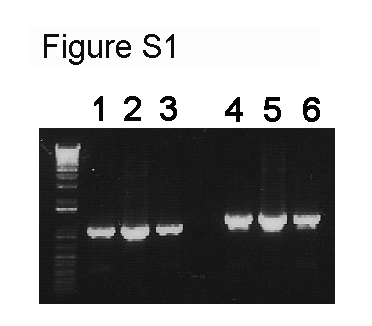

Supplement: Figure S1 — Agarose gel electrophoresis of PCR-amplified cDNA inserts. Nucleic acid sequences of PCR products for vector inserts were verified. Lane 1 and 3: RT-PCR-amplified GFP from total RNA of NIH3T3-GFP; lane 2: Amplified GFP from GFP plasmid (positive control); lane 4 and 6: RT-PCR-amplified MRGD from total RNA of NIH-3T3-MRGD; lane 5: Amplified MRGD from MRGD plasmid (positive control). (TIF) [file pone.0038618.s001.tif]

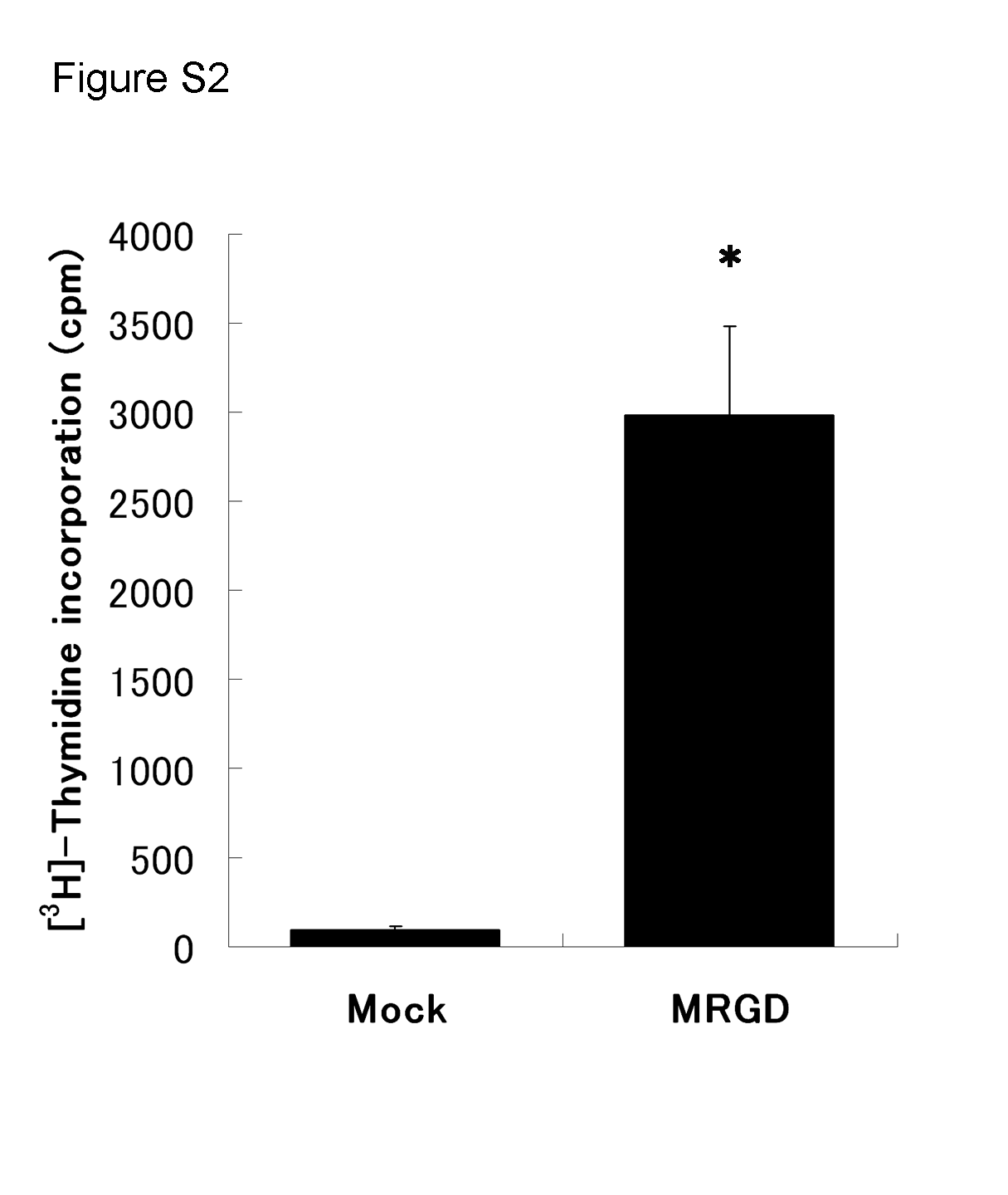

Supplement: Figure S2 — Cell proliferation of NIN3T3-MRGD spheroid measured by [3H]-Thymidine incorporation. [3H]-Thymidine incorporation into the spheroids was measured at 6 days after plating. * indicates p<0.005 (Mann-Whitney U test, 2 tails). (TIF) [file pone.0038618.s002.tif]

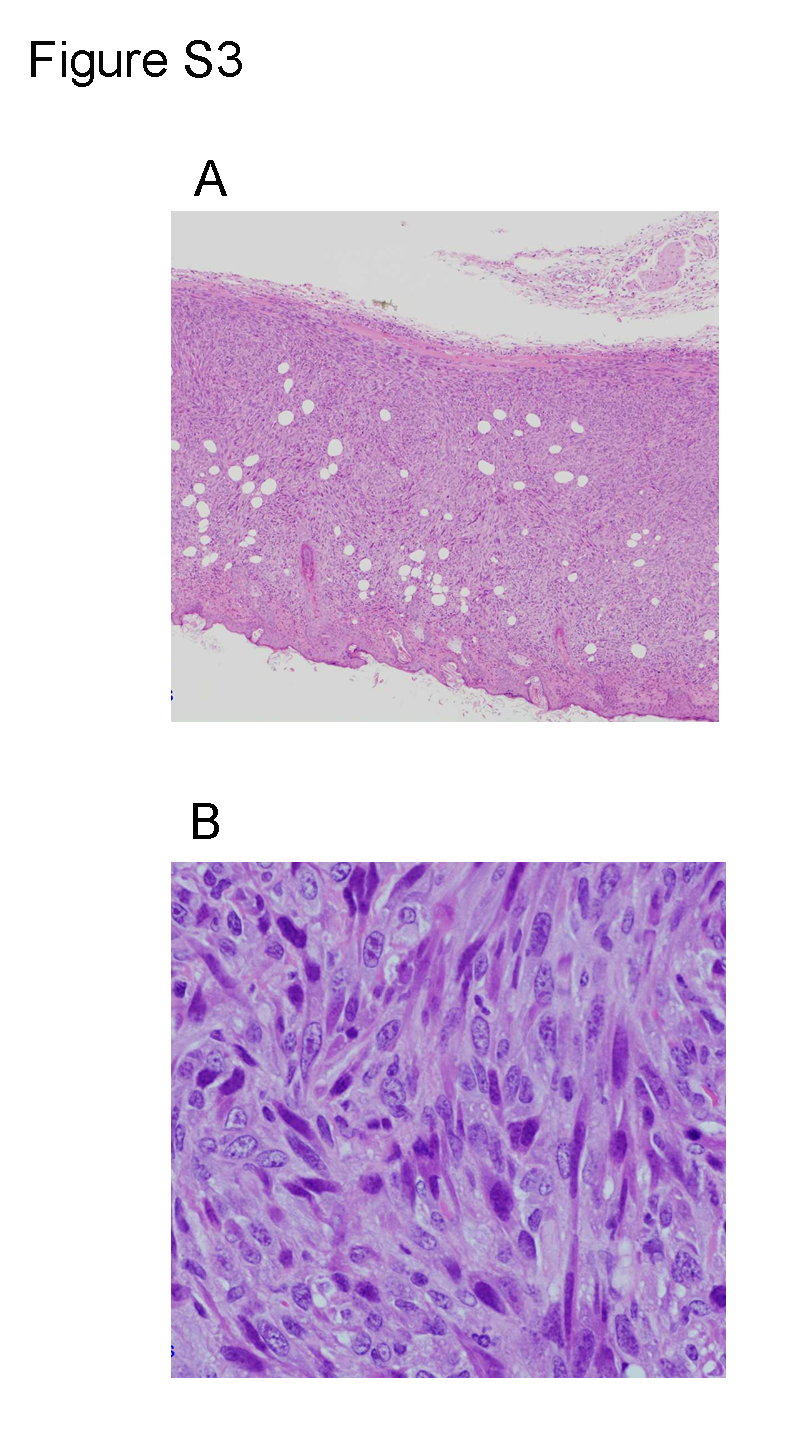

Supplement: Figure S3 — HE staining of NIH3T3-MRGD grafted tissue in nude mouse. The NIH3T3-MRGD clumps on 7 days after inoculation in athymic mice (A, ×40, B, ×800) showed a cellular representative spindle cell tumor tissue type. (TIF) [file pone.0038618.s003.tif]

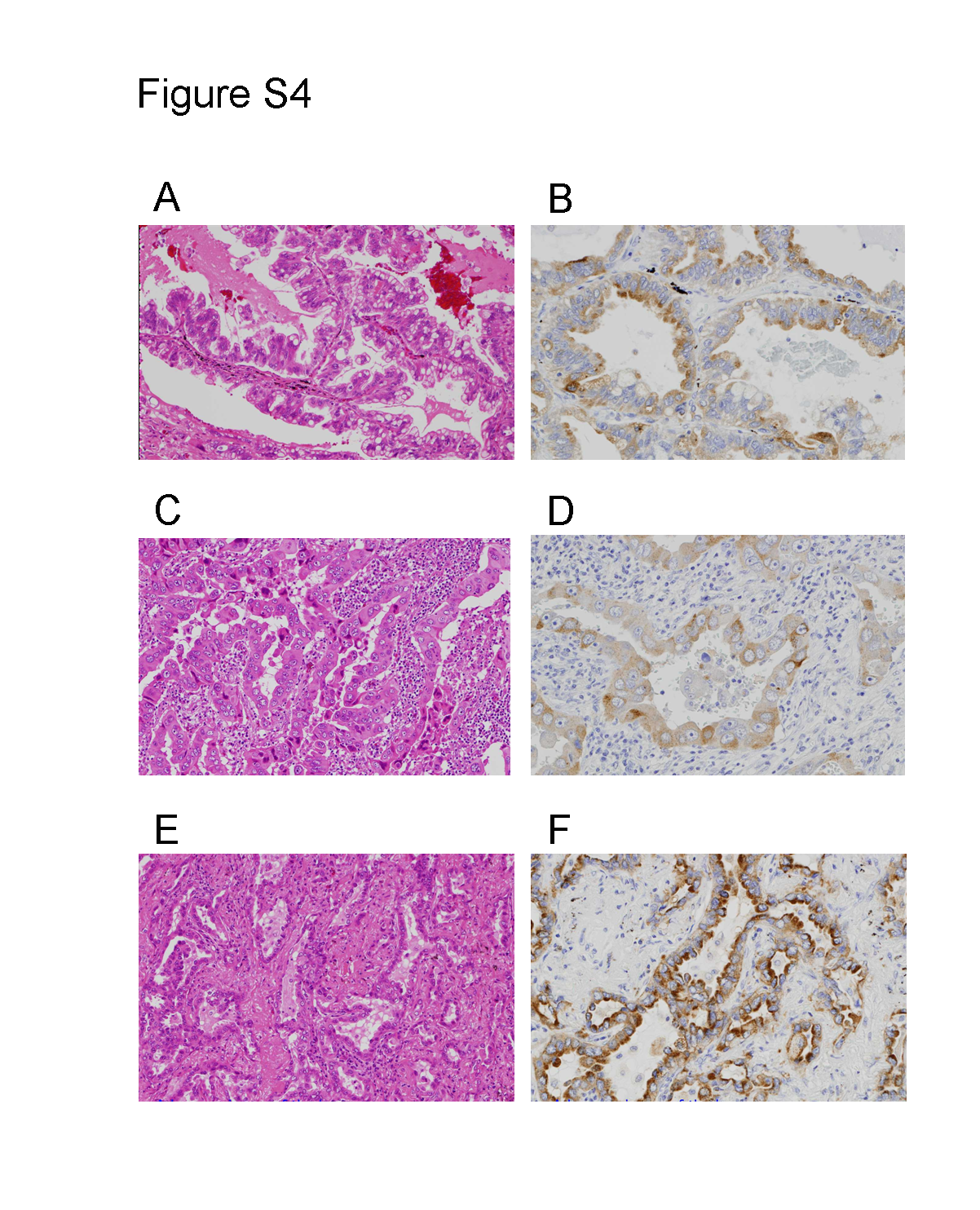

Supplement: Figure S4 — HE and IHC stainings of lung adenocarcinoma samples with anti-MRGD antibody. HE staining (A, C, E) and IHC staining (B, D, F) were performed. These are the examples from three independent patients with lung adenocarcinoma. Patient 1, A, B; Patient 2, C, D; Patient 3, E, F. (TIF) [file pone.0038618.s004.tif]
