# Supplementary material for: MRGD, a MAS-related G-protein Coupled Receptor, Promotes Tumorigenisis and Is Highly Expressed in Lung Cancer
Source: PLoS One. 2012 Jun 8;7(6):e38618. doi: 10.1371/journal.pone.0038618 (PMC3370999; doi:10.1371/journal.pone.0038618)
Supplement: File S1 — Material and Methods of Figure S2. (DOC) [file pone.0038618.s005.doc]

**Supporting Information**

**Materials and Methods S1**

*Thymidine incorporation assay*

To prepare the spheroid growth, see Material and Method in the manuscript. Methyl-[^3^H]-Thymidine (Perkin Elmer Japan, Yokohama, Japan) was diluted to 740 kBq/ml with PBS and 20 μl of diluted methyl-[^3^H]-Thymidine was added to each well in spheroid plates. Spheroids were incubated for 4 hours under 5% CO_2_ at 37°C. After incubation, spheroids were harvested onto a filtermat (Perkin Elmer, Waltham, MA) using a cell harvester (TomTec Imaging Systems, Munich, Germany), and soaked in scintillation solution (Betaplate Scint, Perkin Elmer, Waltham, MA). [^3^H]-Thymidine signals were measured by 1205 Betaplate (Perkin Elmer, Waltham, MA).
